# Auto-recruitment of dorsal lung regions in horses after anaesthesia

**DOI:** 10.1186/2197-425X-3-S1-A682

**Published:** 2015-10-01

**Authors:** M Mosing, AD Waldmann, P Mac Farlane, SH Bohm, SH Iff, R Bettschart, D Bardell

**Affiliations:** Vetsuisse Faculty, Division of Anaesthesiology, University of Zürich, Zürich, Switzerland; Swisstom AG, Landquart, Switzerland; Langford Veterinary Services, University of Bristol, Langford, United Kingdom; CTU Bern, University of Bern, Bern, Switzerland; Vetsuisse Faculty, University of Zurich, Zürich, Switzerland; School of Veterinary Science, University of Liverpool, Leahurst, United Kingdom

## Introduction

Collapse of dependent lung regions and high intrapulmonary shunt fractions are very common in anaesthetized horses. We have observed that horses show breath-holding after recovering from anaesthesia, the purpose of which has not been previously evaluated.

## Objectives

To investigate regional time delays within the lungs during post-anaesthetic breath-holding by electrical impedance tomography (EIT).

## Methods

Intrapulmonary shunt was evaluated at the end of 6 hours anaesthesia in 6 horses. EIT measurements were performed before anaesthesia (BL) and hourly 1 to 6 hours after recovery from anaesthesia. Seven regions of interest (ROI) were defined within the EIT lung region. Times for each specified ROI to reach 50% of maximum inspiratory impedance change (t_filling_) and to remain above 50% (t_inflated_) were determined. Times were then normalised for the total inspiration time and total breath length, respectively.

Linear regression was drawn for time points 1 to 6 and visually checked for significance, using the 95% CI interval of the baseline measurements and the corresponding 95% CI intervals of the linear regression.

## Results

Shunt at the end of anaesthesia was 26 ± 11 %. Compared to BL the five ventral ROIs had a significantly shorter t_filling_ 3 to 5 hours into the post-anaesthesia period. After recovery, t_inflated_ was unchanged in the two most ventral ROIs whilst the more dorsal ROI showed t_inflated_ was extended compared to BL.

## Conclusions

Baseline t_filling_ and t_inflated_ were similar across all ROI. After recovery ventral lung regions showed more rapid filling and emptying, whilst dorsal regions filled more slowly and remained inflated for longer time periods. These findings are consistent with redistribution of air from ventral into dorsal regions during breath-holding period, which may suggest auto-recruitment of atelectic lung regions.

## Grant Acknowledgment

We want to thank the “Stiftung Forschung für das Pferd” for financing this project.

